# Enhancing public health resilience in urban disaster settings: A study protocol on civil-military coordination in Malaysia

**DOI:** 10.1016/j.mex.2023.102456

**Published:** 2023-10-20

**Authors:** Aida Jaffar, Ambigga Krishnapillai, Badrul Hisham Abd Samad, Wan Farizatul Shima Fakuradzi, Nurhan Norris Ma, Halyna Lugova

**Affiliations:** a Primary Care Unit, Faculty of Medicine and Defence Health, Universiti Pertahanan Nasional Malaysia, Wilayah Persekutuan Kuala Lumpur 57000, Malaysia; b Humanitarian Assistance and Disaster Relief Research Centre, Faculty Of Defence Studies And Management, Universiti Pertahanan Nasional Malaysia, Wilayah Persekutuan Kuala Lumpur 57000, Malaysia; c Community Medicine Unit, Faculty of Medicine and Defence Health, Universiti Pertahanan Nasional Malaysia, Wilayah Persekutuan Kuala Lumpur 57000, Malaysia; d Faculty of Medicine and Health Science, University College Sedaya International (UCSI), Bandar Springhill, Mukim Jimah, Port Dickson, Negeri Sembilan 71010, Malaysia

**Keywords:** Public health resilience, Urban disaster settings, Study protocol, Civil-military, Coordination, Malaysia, Document analysis, In-depth interview, Focus group, Interview, Qualitative study, Public health agencies, Qualitative study.

## Abstract

In Malaysia, the increasing frequency and severity of disasters emphasize the urgent need for enhancing disaster management. Given their significant impact on public health and healthcare, effective disaster management becomes a top priority. This study focuses on urban disasters and aims to identify health needs, assess multi-sectorial response gaps, and propose civil-military coordination mechanisms. To achieve this, a qualitative single-case approach will be employed, involving document reviews, in-depth interviews, and focus group discussions with representatives from key governmental agencies responsible for disaster management. The study will specifically concentrate on Kuala Lumpur, the densely populated and commercially active city. Thematic analysis will be used to systematize and verify the collected data, providing comprehensive insights into the current state of civil-military coordination in disaster response and management from stakeholders' perspectives. By examining their perceptions and experiences, the study will identify existing gaps and challenges in civil-military coordination. Ultimately, the findings will contribute to evidence-based policies and strategies aimed at improving disaster management coordination throughout Malaysia.

## Graphical abstract

**Figure:**
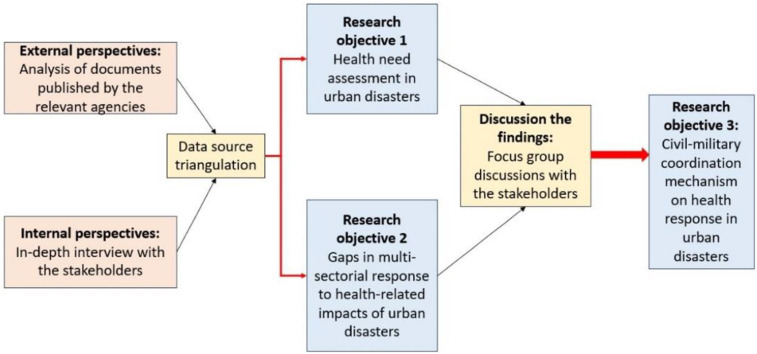

Specifications table

**Table:** 

Subject area:	Environmental Science
More specific subject area:	*A qualitative approach, study protocol on civil-military coordination in disaster response and management*
Name of your method:	Qualitative Study
Name of your protocol:	*Enhancing Public Health Resilience in Urban Disaster Settings: A* *Study Protocol on Civil-Military Coordination in Malaysia*
Reagents/tools:	*In-Depth interview tool utilized in this study for health response to* *urban disaster*
Experimental design:	*Nothing*
Trial registration:	*Not applicable*
Ethics:	• The study will be conducted according to the guidelines of the Declaration of Helsinki. • The study approvals have been obtained from Ethics Committee for Research Involving Human Subjects, University Pertahanan National Malaysia with number JKEP:7/2023.
Value of the Protocol:	• This study protocol significantly contributes by emphasizing the urgent need to enhance disaster management in Malaysia, given the rising frequency and severity of disasters in the country. The research underscores the importance of effective disaster management as a top priority. By specifically focusing on urban disasters, the study addresses a critical aspect of disaster preparedness and response, particularly in densely populated and commercially active areas like Kuala Lumpur. • Employing a qualitative approach, the study will comprehensively explore civil-military coordination in disaster response and management through document reviews, in-depth interviews, and focus group discussions with key governmental agencies. By analyzing stakeholders' perceptions and experiences, the research will uncover prevailing gaps and challenges in this coordination, furnishing valuable insights to enhance the coordination mechanisms. • This study protocol holds significant potential to produce evidence-based policies and strategies for disaster management coordination in Malaysia. Through systematic data analysis using thematic analysis, the research findings will form a solid basis for crafting policies and strategies aimed at addressing identified gaps and challenges in civil-military coordination during disasters. These evidence-based recommendations will strengthen disaster preparedness, response, and recovery efforts, ultimately fostering more resilient and effective disaster management practices across the country.

## Description of protocol

### Background/Introduction

Civil-military coordination is a critical aspect of disaster response that involves collaboration and communication between civilian and military organizations to ensure an effective and efficient response [1]. This coordination is necessary to ensure that resources are deployed effectively, duplication of efforts is minimized, and the needs of those affected are met promptly and appropriately. Malaysia experienced 51 natural disasters from 1998 – 2018, based on a report by Emergency Events Database (EM-DAT) [2]. These include landslides [2], floods [3], tsunamis [4], drought [5] and the Sabah earthquake [6,7]. Natural disasters result in financial damage and loss of precious lives together with disruption in the livelihood of the victims. Effective civil-military coordination depends on establishing sustainable relationships, creating mandatory agreements, preserving transparency, adopting a clear operational viewpoint, and identifying and appreciating organizational cultural differences [8]. Sadly, the poor communication between civilian and military organizations remains a major roadblock to achieving these goals. Additionally, effective interagency coordination is affected by differences in institutional mandates and the chain of command, as well as the absence of a universal guideline or framework for civil-military relations in various contexts [9,10].

### Objectives

The objectives of this study will be (1) to understand the health needs of the population during urban disasters in KL; (2) to explore the gaps in multi-sectoral response to urban disasters in KL; and (3) to develop the optimal civil-military coordination mechanism for responding to health-related challenges in urban disaster situations within KL. We hypothesised that an effective civil-military coordination mechanism on health-related aspects of humanitarian assistance and disaster relief (HADR) can efficiently address the health needs of the population after a disaster and close the gaps in a multi-sectoral response to the health impacts of urban disasters in Kuala Lumpur. The results of the study will provide insights into the challenges and opportunities for improving the response to urban disasters and emergencies in the city. This will also contribute to the development of a framework for civil-military coordination on HADR in KL.

## Method

### Research approach

The research approach will be a qualitative single-case study of the urban area of KL, which is the capital city of Malaysia. Qualitative research aims to explain a phenomenon concerning its nature which includes answering the questions of ‘what’ and ‘how’, as well as to understand a phenomenon from the standpoint of the affected individuals, thus answering the question of ‘why’ [11]. Although the meanings, views and experiences of various stakeholders involved in research are highlighted, no one has a priority for “true knowledge”. The case study method helps in exploring the phenomenon within a particular context through a variety of sources which results in revealing multiple facets of the research phenomenon [12].

KL is one of the most dynamic cities in Southeast Asia, and a vibrant megapolis with a rich history, cultural diversity, and rapid economic growth. Over the years, the city has undergone a significant transformation, with a growing population density due to urbanization, migration, and various development efforts to create a unique identity. Over time, the city of KL has transformed dramatically from a small mining settlement in the late 19th century to a contemporary metropolis with a population of over 7 million. In the year 2000, the population density of the city center, KL city, and KL Metropolitan Region (KLMR) was 6085,7100, or 1052 people per square kilometer. However, by 2004, the population density in the city center had risen to 6710, while it dropped to 6429 in KL city. The rise in population density in KL is due to urbanization, which has attracted migrants seeking better job opportunities, services, and amenities. According to M. Elsayed (2012), the population density in KL increased from 670 in 1980 to 6429 in 2004.

The study is motivated by the growing concern over the heightened risk of disasters in urban areas, particularly in large cities like KL, and the need to examine the efficacy of the existing regulations, policies, and mechanisms related to the public health response to urban disasters and emergencies. The study will focus on the densely populated and commercially active areas of KL.

### Data sources

**Fig. 1:**
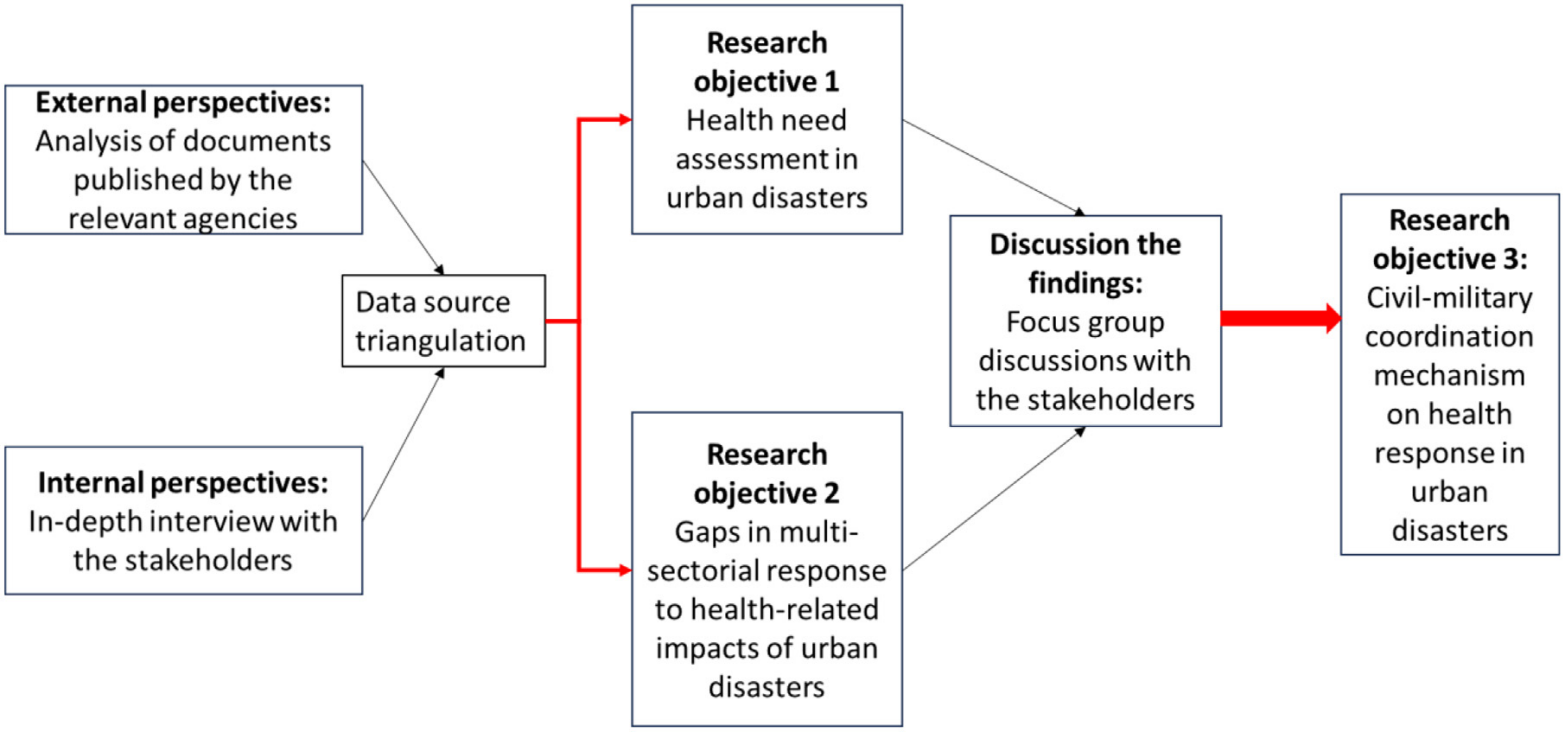
Data source triangulation concerning research objectives (RO).

The methodology of this study involves a thorough analysis and critical evaluation of the existing regulations, policies, and mechanisms related to multi-sectorial public health response, with a specific focus on civil-military coordination. In order to gain a complete understanding of the urban disaster phenomenon and to verify the validity of the findings, we will employ a triangulation strategy. This strategy involves using multiple methods and data sources to collect and analyze information about the research problem, such as document analysis, in-depth interviews, and focus group discussions (FGDs) with representatives from governmental agencies involved in disaster management in KL, including the military. Triangulation helps cross-validate findings and provides a more comprehensive view of the research question [13]. The participants will be carefully selected from agencies identified under the NSC Directive No. 20 to ensure a comprehensive examination of the current situation and experiences related to HADR operations. By employing the triangulation strategy, this study aims to strengthen the validity and reliability of the research findings. This approach will enable cross-validate findings, providing a more robust and solid conclusion to the study (Fig. 1).

## Document analysis

Document analysis is a widely used method in qualitative research, which seeks to study written and/or visual material such as reports, articles, transcripts, and other documents [14], [15], [16]. This method aims to delve deeper into the information found in these documents and gain insight into the perspectives and experiences of those who produced or utilized them. The findings from document analysis can be used to provide valuable information and insights for researchers.

The process of document analysis typically begins with an initial skimming of the material to gain a general understanding. An initial review of the document helps to identify meaningful and relevant passages of text or other data. Subsequently, a more in-depth examination and interpretation of the content is conducted by blending elements of both content analysis and thematic analysis, where information is organized into categories that are relevant to the main research questions. Document analysis is a cost-effective method that enables researchers to obtain empirical data without interfering with or impacting the research participants. Additionally, document analysis combined with the data obtained from interviews and observations can help minimize bias and enhance the credibility of the research findings [15].

This study will conduct a comprehensive review and document analysis of the regulations, policies, and directives concerning health in disaster management in Malaysia, with a specific focus on KL for a year (December 2020- November 2021). Both printed and electronic sources will be utilized, including the identification of the key agencies involved in multi-sectorial responses to urban disasters in the city. The analysis will examine the roles of these key players throughout the phases of the disaster management cycle and address two research questions: (1) What are the health needs of KL residents in different scenarios of urban disasters and emergencies? and (2) What are the gaps in coordination between governmental agencies and the military in health response to urban disasters?

This study will utilize the READ approach (ready, extract, analyze, and distill findings) to document analysis, a systematic method of collecting and gaining information from documents in the field of health policy studies at various levels, such as global, national, and local. The approach involves four steps: (1) preparing the materials, (2) extracting the data, (3) analyzing the data, and (4) summarizing the findings [16]. The data will then be organized in an Excel spreadsheet, with each row representing a document and each column representing a category of information relevant to the study's research questions. This can range from basic information, such as document title, author, and date, to more conceptual categories. The themes will then be imported into thematic coding software, such as NVIVO, for further data extraction [16].

## In-depth interviews

Ensuring the robustness and comprehensiveness of data collected from in-depth interviews (IDIs) is of utmost importance. The number of respondents for the study should be determined based on saturation, which means reaching a point where no new themes emerge from the data. Adding more participants beyond this point does not yield additional information [17]. To achieve this, our team will specifically identify public health responders for urban disaster response in KL, ensuring that relevant respondents are included based on their expertise. This approach guarantees that we capture a wide range of experiences and perspectives.

To prioritize the privacy and comfort of the participants, interview locations will be chosen according to their preferences. Moreover, we will strictly maintain the confidentiality of the participants, taking measures to protect their identities. This includes altering or omitting names and other identifying information during the analysis.

This study will utilize the exponential non-discriminant snowball sampling technique for data collection [18]. This technique allows for the identification of an unlimited number of respondents through key informants. First, the researchers will use purposive sampling to select key informants from each relevant agency. Then, the initial respondents will be asked for their assistance in identifying additional employees from their agency who possess relevant experience, and knowledge, and are willing to participate. This chain referral method is efficient, as it requires fewer resources and enables the researchers to reach the target population promptly.

The data collection process will involve conducting semi-structured interviews estimated to last between 40 and 60 min. The interview guide was designed and copyrighted, which includes several core questions and prompt supplementary questions aimed at exploring the participants' experiences and views on coordinated efforts in response to the public health impacts of urban disasters and emergencies in KL, particularly civil-military coordination (refer appendix). The researchers will use open-ended questions during the interview to allow for flexibility while also recording the interviews using smart pens [19] and observing non-verbal cues. This approach is aimed at gathering more in-depth and nuanced data, as closed or leading questions can restrict the flow of information and limit the data obtained.

## Focus group discussions (FGD)

FGD is a qualitative research method of collecting data through group interaction and discussion. A small, homogeneous group of people are brought together to talk about a specific topic of interest and is facilitated by a moderator. The moderator helps steer the conversation and encourages everyone to participate. FGD is a valuable method for gaining a deeper understanding of attitudes, perceptions, experiences, and beliefs on a specific issue [20]. It quickly produces rich, in-depth data and provides a platform for group members to share their views, elaborate on their opinions, and learn from one another. This method is commonly used in social sciences, health sciences, and other fields where the goal is to comprehend the collective experiences and perspectives of a group.

The aim of this study is to gather comprehensive information about the collaboration and coordination between primary stakeholders of public health response during HADR operations in KL. The FGDs will be centered around topics such as the health risks faced by the KL population and responders in the event of disasters, the state of readiness of healthcare assets and facilities, and potential approaches to enhance the coordination between different agencies in public health to disasters, especially with regard to civil-military coordination.

Attendees for the FGDs will be chosen using a purposeful sampling method to ensure that all relevant stakeholders are included. Invitees include organizations such as the National Disaster Management Agency (NADMA), the Ministry of Health (MOH), the Ministry of defense (MOD), the Department of Social Welfare (JKM), Malaysia Civil defense Department (APM), Royal Malaysia Police (PDRM), Fire and Rescue Department of Malaysia (JBPM) and The Special Malaysia Disaster Assistance and Rescue Team (SMART). The study will conduct two FGDs, each lasting between 60 and 90 min, to gather the necessary data.

The individuals participating in the FGDs will be asked to provide verbal informed consent prior to the session. This will ensure they are fully informed about the purpose and nature of the study and that they are willingly choosing to participate. The FGD will bring together crucial stakeholders involved in HADR operations in KL. The discussions will center on the coordination and responsibilities of organizations during HADR operations, particularly regarding the military's role. To ensure a productive and manageable discussion, the number of participants in the FGD will be limited to 8–10 [20,21]. The researchers will use probing, follow-up, and exit questions to gain a deeper understanding of the topics being discussed. The researchers will moderate the session and record using smart pens [19], and notes will be taken during and after the FGD to capture all important information.

## Data analysis

This study will utilize a thematic analysis approach to systematize and increase the traceability and verification of the data [22]. The textual data produced from the document analysis, and transcriptions of IDIs and FGDs, will undergo a coding process to identify consistent themes. NVIVO software will be utilized to aid in the organization and analysis of the text by counting the frequency of words in the coding phase and helping to validate the findings produced by the researchers.

Thematic analysis of the data from IDIs will be used and common ‘themes’ will be categorized in responses which describe the experiences with regard to multi-sectorial coordinated public health response to disasters in KL. The data will be coded deductively, with the main themes predetermined by the research questions, and sub-themes defined during the analysis. Relationships between the codes will be identified, and the themes and sub-themes will be mapped to demonstrate their relationship.

The data from the FGD will undergo a similar analysis and will be combined with the data from the IDIs to cross-check and validate the findings. Additionally, the findings will be supported by the data obtained from document analysis. Finally, the study will develop and validate a civil-military coordination mechanism on health response to urban disasters in KL with relevant stakeholders.

## Trustworthiness and limitations

Trustworthiness is a crucial aspect of qualitative research that ensures the reliability and validity of the study results. To achieve this, researchers consider four strategies in their studies, including credibility, transferability, dependability, and confirmability [23,24]. In addition to these strategies, this study will comply with the Consolidated criteria for reporting qualitative research (COREQ) checklist for its reporting. The COREQ checklist is a tool designed to enhance the clear and thorough reporting of qualitative research, specifically IDIs and FGDs [25]. It focuses solely on reporting criteria specific to qualitative studies and excludes general criteria that apply to all types of research reports. This comprehensive checklist covers the critical components of study design that should be reported, including information about the research team, study methodology, study context, results, analysis, and interpretations. The inclusion of these criteria within the checklist can assist researchers in accurately reporting crucial aspects of their study. This checklist provides guidelines for transparently reporting the methods and results of qualitative studies.

Noteworthy, this study has limited scope due to the potential unavailability of certain documents, which are classified or restricted for security purposes. Furthermore, health is often viewed as a task exclusive to the healthcare system, rather than a collaborative effort. Unfortunately, many agencies involved in disaster management fail to recognize their role in a holistic public health response to disaster events, instead perceiving healthcare professionals as solely responsible for it.

## Ethical considerations

The privacy and confidentiality of participants in the IDIs and FGDs will be strictly guarded. Participants will be informed before the interviews that their identities will remain confidential and measures will be taken to ensure anonymity during the analysis process, such as changing or omitting personally identifying information and sensitive demographic information. These steps will help build trust with the participants and facilitate open and honest conversations during the data collection process.

If the interviewer or participant feels that the interview is having a negative impact on the participant's emotional state, either party will have the right to interrupt or suspend the interview. This will protect the participant's well-being and avoid undue stress or discomfort. To empower our participants and safeguard their rights, we will be offering them the opportunity to withdraw from the study at any given time without being required to justify their decision. This approach will help to foster a sense of autonomy and control among our participants, without compromising the quality of services they receive.

This study has received ethical approval from the local committee responsible for evaluating and approving research studies, ensuring compliance with both the Declaration of Helsinki and national legislation providing ethical research guidelines and standards.

## CRediT authorship contribution statement

Aida Jaffar and Ambigga Krishnapillai - conceptualization, drafted manuscript, revised manuscript, Badrul Hisham Abd Samad and Nurhan Norris Ma - revised manuscript, Halyna Lugova - conceptualization and revised manuscript, Wan Farizatul Shima Fakuradzi - revised manuscript.

## Availability of data

Data will be made available on request.

## Funding

This research was funded by the Ministry of Higher Education of Malaysia, grant TRGS/1/2020/UPNM/02/1/4.

## Ethical approval

The study will be conducted according to the guidelines of the Declaration of Helsinki. The study approvals have been obtained from Ethics Committee for Research Involving Human Subjects, University Pertahanan National Malaysia with the number JKEP:7/2023.

## Declaration of Competing Interest

The authors declare that they have no known competing financial interests or personal relationships that could have appeared to influence the work reported in this paper.

## Figures and Tables

**Table:** 

Theme	QUESTIONS
**Theme 1** Conceptual understanding of basic terms in disaster management	(1) What do you understand by urban disaster? (2) What do you understand by urban emergency?
**Theme 2** Health risk in disasters: (1) Among population (2) Among responders	(1) Can you describe health risks faced by the population in KL in event of an urban disaster? (2) Can you describe the health risks faced by the responders in an urban disaster?
**Theme 3** Health needs assessment (1) Understanding (2) Experience (3) Challenges (4) Guidelines	(1) What do you understand by health needs assessment in urban disaster in the response phase? (2) Can you share your experience of health need assessment (HNA) in urban disasters? (3) What do you think are the challenges in health need assessment in urban disasters/or any disaster? (4) Have you heard about ASEAN SASOP document and / or SPHERE Project for HNA? (5) What are your views on the ASEAN SASOP document (ARCHProject) for health needs assessment? (6) What are your views on the Sphere Project for health assessment needs? (7) Are you using other documents for HNA in urban disasters?
**Theme 4** Command, Control and Communication: (1) Understanding (2) Challenges (3) Suggestions	(1) What do you understand by command, control and communication system (3Cs) in disaster management? (2) What are the challenges in 3Cs? (3) What are your suggestions to address these challenges?
**Theme 5** Civil-military coordination: (1) Experience (2) Challenges (3) Suggestions	(1) Can you share your personal experiences on coordinated efforts between civilian and military actors in response to public health impacts of urban disasters and /or emergencies in KL? (2) What are the challenges and gaps faced by civilian and military actors in public health response to urban disasters? (3) What are your suggestions to improve civil-military coordination of public health response to urban disasters?
**Theme 6** Health resources: (1) Readiness (2) Strengthening	(1) Can you share your opinion regarding the readiness of the heath resources for the disaster in KL? (2) Can you suggest approaches on how to improve the response of the health system to urban disasters?

## Appendice 1

Questionnaire on in-depth interview for health response to urban disaster in civil-military coordination.
